# Quercetin and Coumarin Inhibit Dipeptidyl Peptidase-IV and Exhibits Antioxidant Properties: In Silico, In Vitro, Ex Vivo

**DOI:** 10.3390/biom10020207

**Published:** 2020-01-31

**Authors:** Anand-Krishna Singh, Pankaj Kumar Patel, Komal Choudhary, Jaya Joshi, Dhananjay Yadav, Jun-O Jin

**Affiliations:** 1 Institute of Life Science, Shri Vaishnav Vidyapeeth Vishwavidyalaya, Indore 452111, India; 2 School of Life Sciences, Devi Ahilya University, Indore, Madhya Pradesh 452001, India; pkjkmr1@gmail.com (P.K.P.); komal.patidar@gmail.com (K.C.); 3 Oral Pathology and Microbiology, Government Dental College, Indore, Madhya Pradesh 452001, India; dr_jayajoshi@yahoo.co.in; 4 Department of Medical Biotechnology, Yeungnam University, Gyeongsan 712-749, Korea

**Keywords:** antiperoxidative, coumarin, diabetes, dipeptidyl peptidase-IV, hyperglycemia, quercetin

## Abstract

Quercetin and coumarin, two naturally occurring phytochemicals of plant origin, are known to regulate hyperglycemia and oxidative stress. The present study was designed to evaluate the inhibitory activity of quercetin and coumarin on dipeptidyl peptidase-IV (DPP-IV) and their antioxidant potential. DPP-IV inhibition assays were performed, and evaluated IC_50_ values of diprotin A, quercetin, coumarin, and sitagliptin were found to be 0.653, 4.02, 54.83, and 5.49 nmol/mL, respectively. Furthermore, in silico studies such as the drug-likeliness and docking efficiency of quercetin and coumarin to the DPP-IV protein were performed; the ex vivo antiperoxidative potential of quercetin and coumarin were also evaluated. The results of the present study showed that the DPP-IV inhibitory potential of quercetin was slightly higher than that of sitagliptin. Virtual docking revealed the tight binding of quercetin with DPP-IV protein. Quercetin and coumarin reduced oxidative stress in vitro and ex vivo systems. We report for the first time that both compounds inhibited the DPP-IV along with antioxidant activity and thus may be use as function food ingredients in the prevention of diabetes.

## 1. Introduction 

Type 2 diabetes mellitus (T2DM) is a rapidly growing metabolic disorder with multiple etiologies leading to chronic hyperglycemia and insulin resistance [1]. The current treatment regimen for T2DM includes several oral hypoglycemic agents (sulfonylureas, biguanides, troglitazone, α-glucosidase inhibitors, thiazolidinediones, and secretagogues) and direct insulin therapy [2,3]. However, these treatments fail to achieve rigorous metabolic control in more than 50% of T2DM cases, necessitating the search for novel antidiabetic agents that mimic or enhance the properties of insulin, as well as protect against diabetic complications. Furthermore, these therapies pose various side effects such as obesity, insulin resistance, hypoglycemia, atherosclerosis, and hormonal imbalance [4]. In the effective treatment of T2DM, obesity, HbA1c, and post-prandial glucose are the factors of prime importance [5,6]. 

Therefore, there is urgency for the development of new therapeutics to manage T2DM. Gut hormone based therapies entail an emerging class of antidiabetic drugs belonging to two categories, glucagon-like peptide-1 (GLP-1) agonists and dipeptidyl peptidase (CD26; DPP-IV) inhibitors [7,8]. To explore and study cost-effective alternative drugs with antidiabetic potential through DPP-IV inhibition [9], we selected coumarin (5,6-benzo-2-pyrone) and quercetin (3,3′,4′,5,7-pentahydroxyflavone), two naturally occurring polyphenolic compounds, present in a wide range of foodstuffs, such as fruits, vegetables, and tea [10]. Coumarin and quercetin have been reported to possess antibacterial, antioxidant, and lipid peroxidation inhibition properties, as well as have positive effects on the key hepatic enzymes of glucose metabolism in T2DM [11,12]. Quercetin has been reported to exert a protective effect against beta-cell damage, by ameliorating hyperglycemia, hyperlipidemia, and insulin resistance in diabetic rats [12].

There has been renewed interest in the development of phytochemicals that can be used as antidiabetic compounds. Flavonoids are naturally occurring phenolic compounds that are widely distributed in the plant kingdom with proven antioxidant properties [13,14]. Therefore, in the present study, based on the available literature and evidence, and our own experience of working in this field, we conducted experiments to confirm our theory that the glucoregulatory mechanism of coumarin and quercetin in T2DM might be mediated through the inhibition of DPP-IV enzyme activity. The antiperoxidative potential of coumarin and quercetin was estimated using β- carotene bleaching assays, inhibition of lipid peroxidation in liver tissue, and erythrocyte haemolysis. A DPP-IV inhibition assay was performed to determine the inhibitory potential of the test phytochemicals. To validate our hypothesis, we conducted molecular docking of coumarin and quercetin with DPP-IV protein and evaluated the criteria of Lipinski’s rule to determine the “drug-likeness” of the prospective drug candidates, with sitagliptin and diprotin A considered as standard DPP-IV inhibitors [15].

## 2. Materials and Methods 

### 2.1. Chemicals

DPP-IV enzyme, Gly-Pro-p-nitroanilide substrate and Coumarin (5,6-benzo-2-pyrone) (C4261) were purchased from Sigma-Aldrich, USA and Quercetin 2-(3,4-dihydroxyphenyl)-3,5,7-trihydroxy-4H-1-benzopyran-4-one dehydrate) RM6194 was obtained from HiMedia Chemicals Pvt. Ltd., Mumbai, India. All the chemicals used in this experiment were of analytical grade, and procured from Merck India Pvt. Ltd. Mumbai, India.

### 2.2. Experimental Animals

Adult male Wistarrats (*Rattusnorvegicus*), weighing 180–220 g, were housed in polypropylene cages (43 × 27 × 25 cm with a floor area of 165.85 cm^2^/animal) and acclimated for a week before experimentation in a 14 h light: 10 h dark in an environment with a temperature of 23 ± 2 °C and humidity under control, with free access to laboratory feed (Gold Mohur feed, Hindustan Lever Ltd., Mumbai, India) and drinking water. Animals were maintained as per the guidelines of the Institutional Animal Ethics Committee (IAEC), the Committee for the Purpose of Control and Supervision of Experiments on Animals (CPCSEA), the Ministry of Environment and Forests, Government of India, New Delhi. The experimental protocol was approved by the IAEC of the School of Life sciences, Devi Ahilya University, Indore. (Registration no. 779/Po/Ere/S//03/CPCSEA).

### 2.3. In Silico and Molecular Docking Studies

The structure of coumarin, quercetin, diprotin-A, and sitagliptin molecule were sketched in the Chemdraw Ultra 6.0 module of Chem. Office 6.0. The sketched molecules were copied and pasted to Chem 3D saved in a mol format, followed by the energy minimization of the structure. The three-dimensional structure of dipeptidyl peptidase IV/CD26 (PDB: 4J3J) was retrieved from the protein data bank [16]. Before the docking of the compound, the protein was optimized and prepared by removing the bound crystal water molecules and adding hydrogen bonds. Explicit hydrogen atoms were created, and bond order, hybridization, and charges were assigned wherever missing. The resulting structure was saved in the PDB format for further study. The molecular docking program Molegro Virtual Docker 5.0 2012 provided a flexible platform for the docking of coumarin, diprotin-A, quercetin, and sitagliptin. Ligand-protein affinity was calculated based on the molecular docking scoring function (MolDock Score) derived from the piecewise linear potential (PLP) scoring functions originally proposed by Gehlhaaret al. [17] and later extended by other researchers [18]. After the ligand was docked, the total energy was minimized using Nelder Mead simplex minimization (using non-GRID force-field and directional H-bonding) [19]. The binding affinity and interaction between the agonist and receptors were evaluated based on internal electrostatic interactions, hydrogen bond interaction, and sp2-sp2 torsions. The optimized compound binding affinity was selected against dipeptidyl peptidase-IV, using the rerank score enzyme (Table 1).

### 2.4. DPP-IV Inhibition Activity 

The DPP-IV enzyme inhibition assay was performed following the protocol described in previously studies [20,21]. In a 96-well microtiter plate, the chromogenic substrate was cleaved by the serine protease DPP-IV, which resulted in the release of 4-p-nitroaniline (pNA), a yellow-colored product. In brief, the DPP-IV inhibition activities of the compounds at various concentrations were determined by measuring the release of 4pNA from an assay mixture containing 20 µL of DPP-IV enzyme, 0.1 M Tris-HCL buffer (pH 8.0), and varying concentration of the test compounds, with sitagliptin and diprotinA. After incubation for 10 min, 50 µL of 2 Mm Gly-Pro p-nitroanilide (substrate) was added. The samples were then incubated for 30 min at 37 °C, and the reaction was stopped by the addition of sodium acetate buffer (pH 4.5). The absorbance was measured at 405nm on a microtiter plate reader. Diprotin A and sitagliptin samples were used as standards. A decrease in DPP-IV enzyme activity was observed as the formation of the pNA yellow coloured product decreased due to enzyme inhibition.

### 2.5. β-Bleaching Inhibition Assay

The antioxidant activity of coumarin and quercetin (analyte) was determined using the β-carotene-linoleate model system [22] with few modifications. In brief, a solution of β-carotene was prepared by dissolving 2 mg of β-carotene in 2 mL of chloroform. Then, 0.2 mL of this solution was pipetted into a 100 mL round bottom flask. The chloroform was removed at 40 °C, followed by the addition of 20 mg of linoleic acid and 200 mg of Tween 40 (polyoxyethylenesorbitanmonopalmitate). The resulting solution was mixed well by shaking, and then 10 mL of distilled water was added, followed by the addition of 40 mL oxygenated water. Aliquots (4 mL) of this emulsion were mixed with 0.2 mL of the analyte at concentrations ranging from 12.5 µg/mL to 200 µg/mL. A control was prepared by adding 0.2 mL of 60% methanol instead of the analyte. Absorbance was measured after 5 min of incubation, at 470 nm at the starting time t = 0. A blank devoid of β-carotene and analyte, and containing linoleic acid, tween 40, and analyte solvent was used for background subtraction. A control containing β-carotene emulsion (4 mL) and analyte solvent (0.2 mL) was also prepared. The mixture was incubated in a water bath at 50 °C for 20 min, and the absorbance was measured at intervals of 15 min. All measurements were performed in triplicate. The percentage inhibition was calculated using the following formula.

% Inhibition = (A_A(120)_ − A_C(120)_ / A_C(0)_ − A_C(120)_) × 100 (1)

where A_A120_ is the absorbance of the analyte after 120 min, A_C120_ is the absorbance of the control after 120 min, and A_C0_ is the absorbance of the control at t = 0 min.

### 2.6. Lipid Peroxidation Inhibition Activity

A male Wistar rat was anesthetized with mild chloroform and sacrificed to obtain liver tissue. A 10% (*w*/*v*) liver homogenate was prepared in phosphate-buffered saline (PBS; 0.1 M, pH-7.4) using a simple homogenizer. Varying concentrations (25–400 μg/mL) of coumarin, quercetin and ascorbic acid were incubated with 1 mL of the rat liver homogenate. The reaction was initiated by the addition of 0.1 mL of FeSO_4_ solution (25 μM), 0.1 mL of ascorbate (100 μM), and 0.1 mL of KH_2_PO_4_ (10 mM); the final volume was made up to 3 mL with distilled water and incubated at 37 °C for 1 h. Then, 1 mL of 5% trichloroacetic acid (TCA) and 1 mL of 1% thiobarbituric acid (TBA) were added to the reaction mixture, and the tubes were boiled for 1 h in a water bath followed by centrifugation at 3500 rpm for 10 min. The extent of lipid peroxidation was evaluated by estimating the thiobarbituric acid reaction substances (TBARS) level by measuring the absorbance at 532 nm [23].

% inhibition = (A_C_ − A_S_/ A_C_) × 100 (2)

### 2.7. Erythrocyte Haemolysis Inhibition Activity

The antiperoxidative potential of coumarin and quercetin was determined by the inhibition of erythrocyte hemolysis [24] with a slight modification of the given method. A Wistar male rat was ether-anesthetized, and its heart was punctured to collect its blood in a beaker containing sodium citrate (3.8%). Erythrocytes, were separated from the plasma, and the buffy coat, was washed three times with 10 mL of PBS and centrifuged at 1500 rpm for 5 min at room temperature. Then, 2 mL of the erythrocyte suspension (4%) in PBS was added to 1 mL of the analyte or standard (25–1000 μg/mL), followed by the addition of 0.5 mL of freshly prepared 100 mM H_2_O_2_. The final volume was adjusted to 4 mL by the addition of PBS. The reaction mixture was shaken gently during incubation at 37 °C for 3 h. The control contained all the constituents as above, except the analyte or standard. The reaction mixture was diluted with 8 mL of PBS and centrifuged at 3000 rpm for 10 min; the absorbance of the supernatant was measured at 540 nm on a UV–VIS Spectrophotometer. The percentage of the hemolysis inhibition was calculated using the following formula:

% hemolysis inhibition = [(A_C_ − A_S_)/A_C_] × 100 (3)

where A_S_ is the absorbance of the sample containing coumarin, quercetin or standard, and A_C_ is the absorbance of the control sample containing no analyte. The assays were carried out in triplicate, and the results are expressed as mean values ± standard error. L-ascorbic acid was used as a standard.

### 2.8. Statistical Analyses

Data are expressed as mean ± S.E.M. Analysis of variance (ANOVA) followed by a post-hoc Newman–Keuls multiple comparison test were used for the statistical evaluation of data using a trial version of Prism 4 for Windows (Graph pad Software, Inc., La Jolla, CA, USA). The paired two-tailed Students‘ *t*–test was used to analyze the significance between the sets of paired means. Data were considered statistically significant at *p* < 0.05.

## 3. Results

### 3.1. In Silico Studies

**Figure 1:**
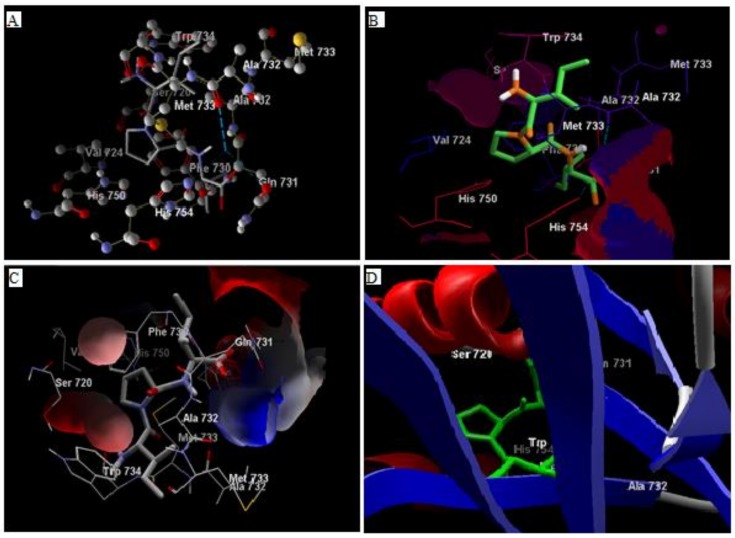
The hydrogen bonding (**A**), hydrophobic binding (**B**), electrostatic binding (**C**), and secondary protein interaction (**D**) of diprotin-A and dipeptidyl peptidase-IV protein.

**Figure 2:**
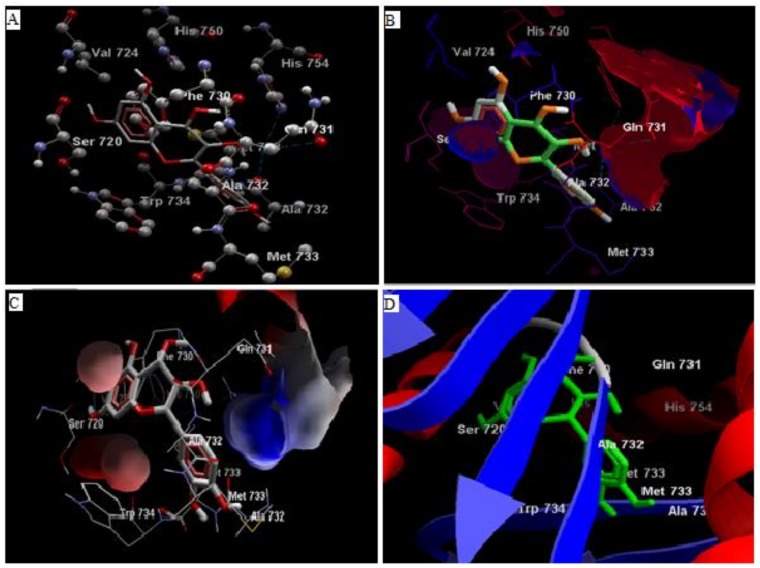
The hydrogen bonds (**A**), hydrophobic binding (**B**), electrostatic binding (**C**), and secondary protein interaction (**D**) of quercetin and dipeptidyl peptidase-IV protein.

**Figure 3:**
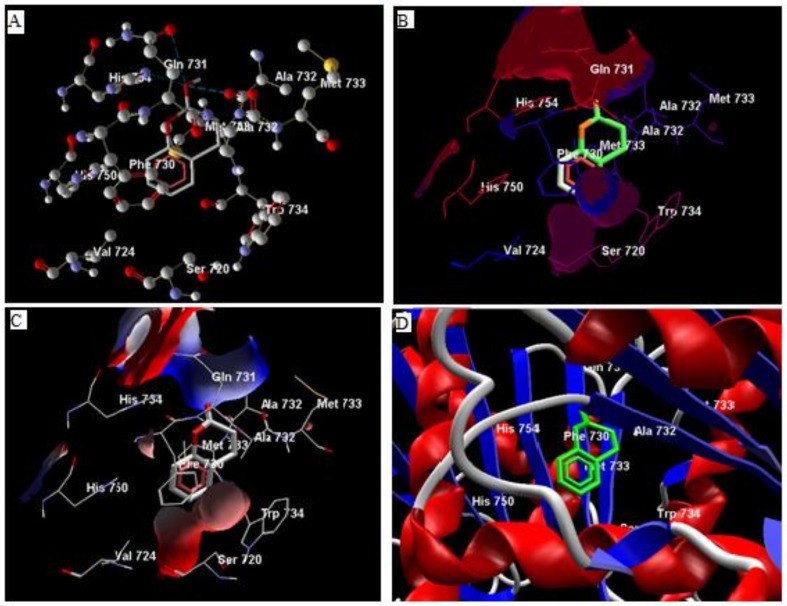
The hydrogen bonds (**A**), hydrophobic binding (**B**), electrostatic binding (**C**), and secondary protein interaction (**D**) of coumarin and dipeptidyl peptidase-IV protein.

**Figure 4:**
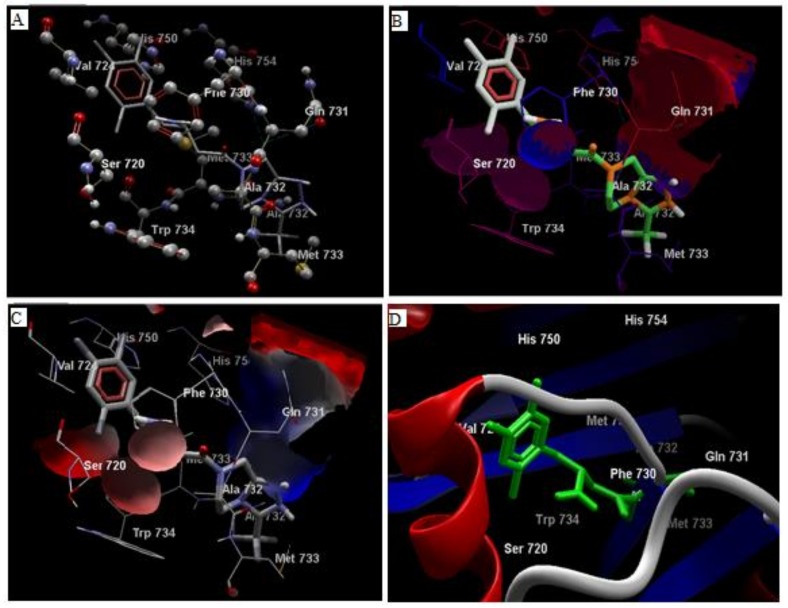
The hydrogen bonds (**A**), hydrophobic binding (**B**), electrostatic binding (**C**), and secondary protein interaction (**D**) of sitagliptin and dipeptidyl peptidase-IV protein.

Comparative analysis of the structural components of quercetin and coumarin revealed that the functional groups of both phytochemicals interacted with the agonist binding site of the DPP-IV enzyme. The confirmation of diprotin A is shown below (Table 1, Figure 1). Quercetin interacts with the conformer and forms 5 hydrogen bonds with the hydroxyl groups of Val 738, Ser 720, Tyr 700, Ala 732 and Met 733 (Figure 2). Coumarin interacts with the conformer and forms 2 hydrogen bonds with the C=O groups of Gln 731 and Ala 732 (Figure 3). Sitagliptin interacts with the conformer and forms 2 hydrogen bonds with the five-membered nitrogen ring system of His 754 and Ala 732 (Figure 4).

### 3.2. Analysis of Ligand Binding Affinities

The MolDock score, re-rank score, and hydrogen bond interaction showed a varying pattern and were measured as −54.17, −48.50, and −3.180, respectively, for coumarin; −107.70, −6.68, and −6.58, respectively, for diprotinA; −85.49, −72.54, and −11.21, respectively for quercetin; and −108.25, −77.68, and −2.29, respectively for sitagliptin (Table 1).

### 3.3. DPP-IV Inhibition Activity 

The results of this experiment revealed that diprotin-A, quercetin, coumarin, and sitagliptin inhibited DPP-IV activity. Quercetin inhibited DPP-IV enzyme activity noticeably more than coumarin and sitagliptin. The IC_50_ values for the DPP-IV inhibition of diprotin-A, quercetin, coumarin, and sitagliptin were 0.653, 4.02, 54.83, and 5.49 nmol/mL, respectively (Table 2).

### 3.4. β-Carotene Bleaching Inhibition

**Figure 5:**
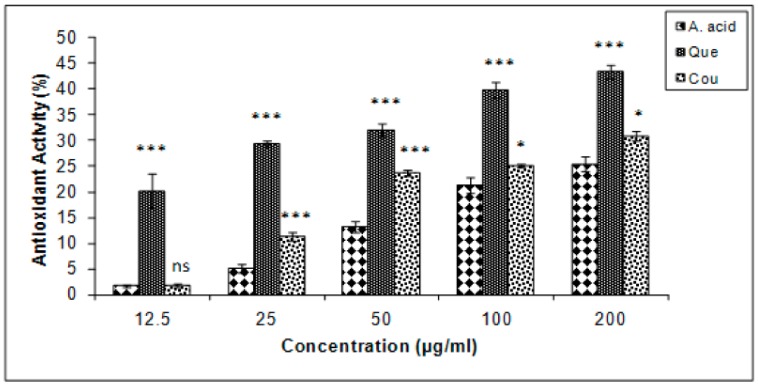
β carotene bleaching inhibition (%) in quercetin and coumarin as compared to the control; ascorbic acid. Each vertical bar represents the mean ± S.E.M. (n = 3), *** *p* < 0.001, ** *p* < 0.01 and * *p* < 0.05 as compared to the respective control values. A. acid, ascorbic acid; Que, quercetin; Cou, coumarin.

Quercetin exhibited the highest levels of β-carotene bleaching inhibition in the linoleate system, followed by coumarin and ascorbic acid (Figure 5). The results are expressed in terms of % inhibition. The IC_25_ of ascorbic acid, quercetin, and coumarin, were 173.5, 2.71, and 127.66 µg/mL, respectively.

### 3.5. Hepatic Lipid Peroxidation Inhibition

**Figure 6:**
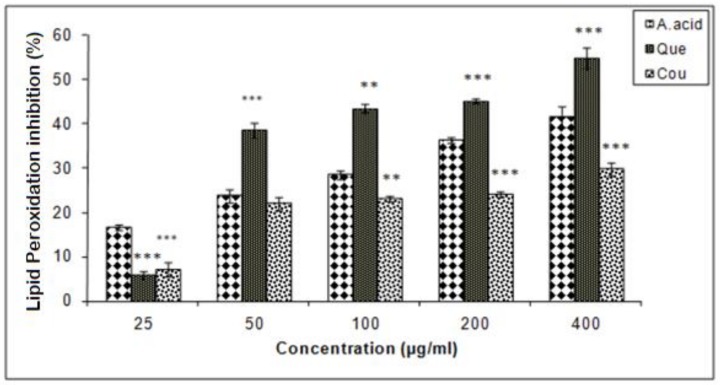
Hepatic lipid peroxidation inhibition activities of quercetin and coumarin as compared to the control; ascorbic acid. Each vertical bar represents the mean ± S.E.M. (n = 3). *** *p* < 0.001, ** *p* < 0.01 and * *p* < 0.05 as compared to the respective control values. A. acid, ascorbic acid; Que, quercetin; Cou, coumarin.

Data of this experiment suggested that quercetin exhibited a higher inhibition of lipid peroxidation as compared to ascorbic acid and coumarin. The IC_25_ values of coumarin, quercetin and ascorbic acid in lipid peroxidation inhibition were 248.5, 12.5, and 75 µg/mL respectively (Figure 6).

### 3.6. Erythrocyte Hemolysis Inhibition 

**Figure 7:**
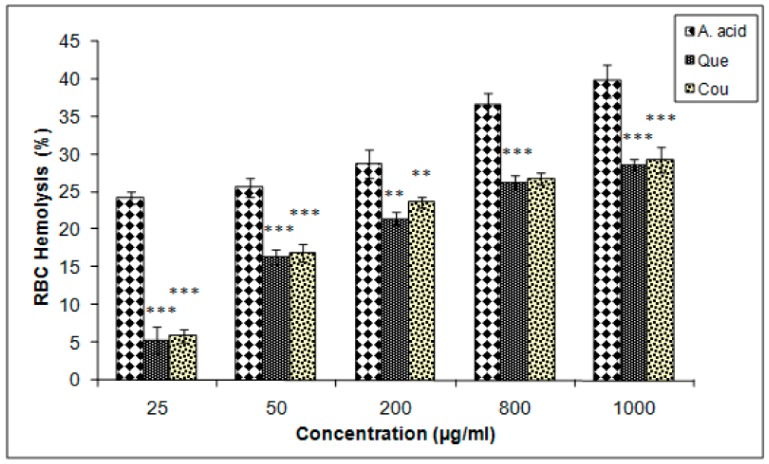
Erythrocyte haemolysis inhibition efficacies of quercetin and coumarin as compared to the control; ascorbic acid. Each vertical bar represents the mean ± S.E.M. (n = 3). *** *p* < 0.001, ** *p* < 0.01 and * *p* < 0.05 as compared to the respective control values. A. acid, ascorbic acid; Que, quercetin; Cou, coumarin.

The membrane-stabilizing potentials of both quercetin and coumarin were almost equal but lower than that of ascorbic acid, as determined by the erythrocytes hemolysis inhibition assay. The IC_25_ values of coumarin, quercetin, and ascorbic acid were 717.5, 737.05, and 12 µg/mL, respectively (Figure 7).

## 4. Discussion

The phytochemicals of plants often play important roles in antioxidants and anti-inflammatory agents; phytochemicals have been found to possess a wide range of activities that may help in protection against chronic diseases [25]. The results of this study demonstrated that quercetin and coumarin significantly inhibited DPP-IV enzyme activity at a level comparable with the standard inhibitor, diprotin-A. DPP-IV is a serine protease that removes X-Pro and X-Ala di-peptides from the penultimate position of the N-terminal end of peptides and proteins [26,27], and is reported to be the primary activator of the incretin hormones GLP-1 and gastric inhibitory peptide (GIP) [28]. DPP-IV inhibitors are safe and effective molecules for T2DM treatment. This study elucidates the potential mechanism to treat diabetes by quercetin and coumarin that inhibited the DPP-IV enzyme activity.

While DPP-IV rapidly inactivates GLP-1 and GIP, the inhibition of DPP-IV prolongs and enhances the activity of endogenous incretins (GLP-1 and GIP), which in turn helps to stimulate prandial insulin secretion and lowers of blood glucose levels. DPP-IV inhibitors represent a unique approach in the treatment of T2DM without any known side effects. This study also discusses the DPP-IV inhibitory potential of quercetin and coumarin, along with their antiperoxidative potential. The active principles of some plants, such as phytochemicals, inhibit DPP-IV enzyme activity [29]. Based on some findings, it is believed that DPP-IV level increased in diabetic condition and associated with long-term exposure to high levels of glucose [30]. The significant reduction in the incretin effect has been known to decrease the circulation level of GLP-1, which may be due to an increase in degradation by DPP-IV that leads to a decline in the mass and size of β-cell in the pancreas [31].

Comparative examination of the active principles of quercetin and coumarin with sitagliptin, a synthetic DPP-IV-based antidiabetic drug, revealed that quercetin and coumarin might utilize in the treatment of diabetes through DPP-IV inhibition or insulin stimulation. However, in previous reports, quercetin may reduce the risk of T2DM; by inhibition of glycogen phosphorylase which inhibited glycogenolysis, reduced hepatic glucose production [32,33]. Quercetin and rutin reduced glucose absorption from small intestine by inhibition of α- amylase and α- glucosidases to lowering the blood glucose level [34,35,36]. Coumarin showed antidiabetic activity by stimulating the insulin production from β-cell in pancreas by regulating the glycolysis and inhibition of gluconeogenesis [37]. Nevertheless, till date none of the report suggested that DPP-IV based inhibitor mechanism of these two bioactive compounds.

The compounds quercetin, coumarin and diprotin-A candidate drug molecule as per the Lipinski rule of drug-likeliness. As the compounds have less than 500 dalton molecular weight, high hydrophilicity, less hydrogen donor or acceptor [20]. Data from docking simulations were used to determine the binding orientation and molecular interactions of quercetin, coumarin, and sitagliptin, diprotin-A to DPP-IV enzyme suggested that quercetin and diprotin-A compounds occupied the same ligand-binding domain of DPP-IV with similar orientation due to their molecular shape similarity [20,38]. Docking outcomes from the referred site on a single chain of DPP-IV (4J3J) suggested that coumarin interacts with the two hydrogen bonds of the C=O group of Gln 731, and Ala 732 with the receptor. Hydrophobic interaction is observed between Gln 731, Ala 732, and Phe 731 while electrostatic interactions are observed between Gln 731, Ala 732, and Phe 731 (Figure 3) [39].

Interestingly, a slight variation was observed in the electrostatic pattern of quercetin compared to sitagliptin due to the difference in the hydrogen-bonding pattern. A common interaction was observed between Ser720, Ala732, Met733, Gln731, and Phe730 when coumarin lacked its hydrogen donor functions at the backbone structure. The quercetin interaction showed 50–60% similarity to that of sitagliptin [40].

Hyperglycemia induces the production of free radicals and promotes oxidative membrane damage, which leads to cellular instability. Therefore, it is thought that hyperglycemia in diabetes increases oxidative stress and causes more psychological damage in T2DM than under normal conditions. The use of oxidative stress markers as prognostic predictors may help in the development of better therapeutic strategies for the management of T2DM [41,42].

In the present study, the antioxidant activities of the phytochemicals quercetin and coumarin were tested for their possible antiperoxidative potentials. The antioxidant activity of carotenoids is based on the formation of carotenoids radicals adducts with free radicals from the linoleic acid linoleate system [43]. Phenolic-enriched extracts possessing anti-inflammatory, antiviral, antibacterial, antifungal, and anti-allergic properties have been reported to confer a wide range of physiological and health benefits [44]. Bioactive compounds show promise in reducing the level of free radicals as well as the severity of diabetic complications and many other chronic metabolic diseases. Flavonoids in the human diet may reduce the risk of various cancers as well as prevent menopausal conditions [45]. Quercetin and coumarin exhibited a dose-dependent pattern in regulating the generation of free radicals. It is apparent that quercetin and coumarin reduced the extent of β-carotene bleaching by neutralizing linoleate and other free radicals in the radical system; quercetin was the most effective, with an IC_25_ of 2.71 µg/mL. The antiperoxidative potential is attributed to the aromatic structure, which quenches the free electrons significantly [46].

Membrane lipids are major targets for cellular damage induced by reactive oxygen species (ROS) [47]. Plant-derived active principles have been reported to inhibit ROS levels in living systems [48]. The findings on isolated hepatocytes treated with quercetin or coumarin corroborated this fact, as both phytochemicals reduced lipid peroxidation. The reduction of lipid peroxidation might be attributed to the antioxidant activities of the plant extracts [49].

The erythrocyte inhibition assay revealed that quercetin and coumarin inhibited (28.68 ± 0.78% and 29.31 ± 1.72%, respectively) RBCs membrane lysis to similar extents, exhibiting the membrane-stabilizing potential of both active principles. The reduction capacity of a compound is a significant indicator of its potential antioxidant activity [50]. Hydrogen peroxide interacts with hemoglobin and other intracellular moieties and initiates a series of reactions indicating that hydrogen peroxide could cross the RBC membrane and form ferryl radicals that act on other intracellular moieties [51,52]. Therefore, inhibition of one or more of these events may explain the mechanisms by which quercetin and coumarin inhibit H_2_O_2-_induced RBC hemolysis. Thus, further studies are required to provide additional evidence that strengthens the claim that plant bioactive compounds maybe a potential source of antioxidant-based therapies and that DPP-IV inhibitors may have therapeutic potential for T2DM [53]. The study has some limitations, such as we did not determine the pharmacokinetics and pharmacodynamics experiments to further evaluate the actual biological actions of drugs. The study also lacks in vivo model experiments, which is needed to explain the antidiabetic action of quercetin and coumarin in animals or humans. Hence, we are unable to produce the pharmacokinetic parameters of absorption, distribution, breakdown, and excretion of the drug in the study. From this study, we shaded a possible mechanism to treat diabetes by quercetin and coumarin, which inhibited the DPP-IV.

## 5. Conclusions

Based on the results of this study, we concluded that bioactive compounds from plants have the potential to act as DPP-IV inhibitors, leading to glucose homeostasis, and can be used in the development of novel therapeutic strategies for the treatment of diabetes. DPP-IV plays a vital role in inhibiting the gut hormone GLP-1 and GIP. These incretin hormones regulate insulin from β- cells in the pancreas. We found that quercetin and coumarin were able to bind with the active site of the DPP-IV enzyme in a manner that was similar to diprotin-A and sitagliptin. In addition to their DPP-IV inhibitory activities, they also exhibited a cytoprotective potential. Nevertheless, the conclusion from this analysis provides the resource to carry on further investigations, to develop quercetin and coumarin as naturally occurring bioactive compounds with DPP-IV inhibitory activity and antioxidant activity.

## Figures and Tables

**Table 1:** The MolDockscore, rerank score, and hydrogen bond interaction energy of the different molecules with Dipeptidyl peptidase-IV.

S.No	Name of Molecule	Mol Dock Score	Rerank Score	H Bond
1	Diprotin-A	−107.70	−6.68	−6.58
2	Quercetin	−85.49	−72.54	−11.21
3	Coumarin	−54.17	−48.50	−3.180
4	Sitagliptin	−108.25	−77.68	−2.29

**Table 2:** DPP-IV enzyme Inhibition activity of Diprotin-A, Quercetin, Coumarin, and Sitagliptin.

Sample	Concentration (nmol/mL)	Inhibition (%)	IC50 (nmol/mL)
Diprotin-A (Ile-Pro-Ile) Positive control	0.19	22.72	0.653
	0.37	40.61	
	0.73	62.82	
	1.46	74.36	
Coumarin	3.42	16.91	54.83
	13.68	28.39	
	27.37	47.64	
	136.85	62.75	
Quercetin	1.65	21.93	4.02
	6.62	58.15	
	13.23	80.88	
	66.17	84.09	
Sitagliptin	3.32	48.6	5.49
	49.10	77.9	
	98.20	81.4	
	147.32	86.4	
	196.41	90.4	
	245.51	95.7	
